# Securing Maternal Identity: Halachic Concerns in the Age of Mitochondrial Replacement Therapy

**DOI:** 10.5041/RMMJ.10587

**Published:** 2026-10-26

**Authors:** John D. Loike, Rabbi Tzvi Flaum, Alan Kadish

**Affiliations:** 1 Professor of Biology and Bioethics, Touro University, New York, NY, USA; 2 Professor of Judaic Studies and Mashgiach Ruchani, Lander College for Women, Touro University, New York, NY, USA; 3 President, Touro University, New York, NY, USA

**Keywords:** Bioethics, biparental MRT, halacha of parenthood, religious status of generating embryos from multiple parents

## Abstract

Mitochondrial replacement therapy (MRT) encompasses assisted reproductive technologies designed to prevent the transmission of pathogenic mitochondrial DNA (mtDNA) mutations by combining the DNA in the nucleus (nuclear genome) of the intending parents with another woman’s enucleated egg that provides healthy donor-derived mtDNA. The use of advanced genomic engineering in MRT raises distinct ethical and halachic considerations regarding the ontological status of embryos containing genetic material from more than two individuals. This paper advances an original theoretical protocol, termed biparental mitochondrial replacement therapy (Bi-MRT), that mitigates the central halachic concerns associated with multi-parent embryos. In this protocol, future advances in stem cell technology could be employed to generate an enucleated gamete-like cell containing healthy mitochondria from the husband. The meiotic spindle–chromosome complex from the wife’s oocyte could then be transplanted into this reconstituted gamete, which could be subsequently fertilized with the husband’s sperm for IVF. If technically feasible and developmentally competent, the resulting embryo could potentially be transferred to the wife for gestation. By combining the wife’s nuclear genetic contribution, gestation, and parturition with the husband’s paternal DNA, this framework isolates the third-party mitochondrial contribution as a non-maternal factor, thereby reinforcing the wife’s singular claim to halachic maternity. Biparental MRT therefore represents a theoretical framework that could potentially reduce some halachic concerns associated with conventional MRT, but substantial scientific and safety barriers remain before any clinical application could be contemplated.

## INTRODUCTION

Mitochondrial DNA (mtDNA) genetic disorders arise from defects in the production of ATP. Pathogenic mtDNA mutations disproportionately affect high-energy-demand tissues, resulting in clinical manifestations such as cardiomyopathy, progressive neuromuscular degeneration, vision loss, and premature mortality.^1^,^2^ Historically, women carrying pathogenic mtDNA variants have faced several difficult reproductive and ethical dilemmas.^3^ For example, should a woman who is herself healthy have an ethical or halachic obligation to alter her reproductive choices because of a genetic risk to a future child? In addition, is it morally acceptable to create multiple embryos knowing that some may be discarded because they contain high levels of mutant mtDNA? While several therapeutic approaches have been examined,^4^ mitochondrial replacement therapy (MRT) has emerged as a reproductive strategy intended to markedly reduce transmission of pathogenic mtDNA variants, thereby allowing these women to give birth to healthier offspring. Mitochondrial replacement therapy (often described in the media as producing “three-parent” offspring) substantially reduces the risk of transmitting severe maternally inherited mitochondrial disease, but it does not eliminate ethical questions concerning embryo creation and disposition, germline transmission, procedural safety, and the long-term consequences of mitochondrial manipulation.

Three principal MRT approaches have been investigated: spindle transfer, pronuclear transfer, and polar body transfer. These MRT approaches are at different stages of development, ranging from preclinical investigation to limited clinical application.^5^ We focus on the spindle transfer model. This protocol involves the isolation of the meiotic spindle apparatus containing the maternal nuclear chromosomes from an unfertilized oocyte, followed by its transfer into an enucleated donor oocyte containing healthy mitochondria. The reconstructed oocyte is subsequently fertilized via intracytoplasmic sperm injection using the sperm from the intended father. If development proceeds normally, this approach may result in an embryo, and potentially a child, with substantially reduced transmission of the maternal pathogenic mtDNA variant. The use of MRT is also being investigated as a potential fertility-enhancing intervention for couples experiencing age-related infertility, although clinical research in this area remains preliminary.^6^

A second approach, called pronuclear transfer, has been used in the United Kingdom (UK) to generate healthy children.^7^ It involves transferring pronuclei from a fertilized zygote (generated *in vitro* from the intended parents) into an enucleated donor zygote containing healthy mitochondrial DNA. Because this procedure requires the creation and subsequent destruction of a donor zygote, ethical concerns have been raised regarding the moral status of the embryo that might otherwise be frozen, used for research, or discarded.

The third approach is called first polar body transfer. The process begins by collecting a mature oocyte from a woman carrying pathogenic mtDNA mutations; this oocyte has already extruded a polar body containing the mother’s nuclear chromosomes but contains little cytoplasm and few mitochondria. Next, a healthy donor oocyte is obtained, and its own nuclear genetic material is carefully removed, leaving behind cytoplasm with normal mitochondria. The maternal polar body, which contains the mother’s chromosomes, is then microsurgically transferred into the enucleated donor oocyte so that the reconstructed egg now possesses maternal nuclear DNA within donor cytoplasm containing healthy mitochondria. After reconstruction, the egg is fertilized with paternal sperm via intracytoplasmic sperm injection. If normal embryonic development is achieved, the resulting embryo could theoretically be considered for embryo transfer.

The UK became the first nation to formally legalize MRT in 2015. In 2025, investigators at Newcastle University reported 8 clinical pregnancies in 22 pronuclear transfer patients, resulting in 8 live births.^7^ Genetic analyses demonstrated that maternal pathogenic mtDNA was largely undetectable, and all infants were reported to be basically healthy at birth. These findings demonstrate that MRT could markedly reduce mutant mtDNA transmission and produce apparently healthy offspring.

The use of MRT raises a broad spectrum of ethical and halachic questions that extend well beyond its medical benefits. This paper deliberately narrows its focus to the determination of halachic maternity and paternity. We propose a theoretical modification of this technology, termed biparental MRT (Bi-MRT), which eliminates the need for a third-party mitochondrial donor by utilizing healthy mtDNA derived from the husband. By restricting all genetic contributions to the married couple, Bi-MRT simultaneously preserves the wife’s nuclear genetic contribution, gestation, and parturition. This paradigm shift in biotechnology could significantly reduce uncertainty regarding halachic parenthood without introducing a third genetic contributor.

## SECULAR BIOETHICAL CHALLENGES ASSOCIATED WITH MRT

Mitochondrial replacement therapy raises several general ethical concerns, including the ethics of human subject research, procedural safety, protection of donor anonymity, obligations of disclosure, and the application of principles of distributive justice.^8^ In addition, because donor mitochondria are maternally inherited, MRT can constitute a form of heritable human germline modification when used to produce female offspring, potentially transmitting the introduced mitochondrial genome from the mtDNA donor to subsequent generations.^9^ Critics argue that accepting MRT could lead to more contentious germline modifications, including eugenic applications, and raise concerns about inappropriate intervention in human evolution.^10^,^11^

## HALACHIC CHALLENGES

Mitochondrial replacement therapy has been regarded in contemporary rabbinic literature as a potentially permissible therapeutic intervention that may enable women carrying pathogenic mitochondrial DNA mutations to bear healthy offspring.^12^ Nevertheless, MRT raises a broad spectrum of halachic concerns, including questions of maternal and paternal identity, *kehuna* [priestly] and *leviyah* [Levite] lineage, embryo manipulation, participation in experimental clinical trials, and potential implications for the mitzvot of *pru u’rvu* [be fruitful and multiply], *yibum* [levirate marriage], and *pidyon ha-ben* [redemption of the firstborn son], as well as concerns regarding prohibited familial relationships and *mamzerut* [the halachic status of offspring from certain prohibited unions]. These important issues warrant separate analysis and are beyond the primary scope of this paper. Instead, this paper focuses on whether mitochondrial donation introduces a third genetic contributor whose involvement may affect the determination of halachic maternity.

### Motherhood Status

Jewish status is transmitted matrilineally according to normative halacha.^13^–^17^ However, the advent of *in vitro* fertilization, gamete donation, and gestational surrogacy has raised complex questions regarding the definition of halachic motherhood. The central question is whether halachic motherhood is established by providing the egg, carrying the fetus during gestation, or giving birth.

When these criteria are divided among two or more women, a halachic *safek* [doubt] regarding motherhood may arise, requiring many halachic authorities to adopt stringent positions regarding incest prohibitions and Jewish status.^12^ For example, if mtDNA is deemed sufficient to confer aspects of halachic motherhood or Jewish status, then an offspring conceived using the mtDNA of a non-Jewish donor might be required to undergo conversion *misafek* [because of uncertainty regarding the child’s Jewish status] (see Table 1).^18^–^24^

**Table 1:** Halachic Approaches to Defining Motherhood in Assisted Reproduction.^18^,^19^

Approach/Criterion	Main Position	Representative *Poskim*/Sources
Genetic/Ovum Source	Maternal status is determined primarily by the woman who provides the ovum and genetic material from which the embryo is formed; gestation alone does not displace genetic maternity.	Rabbi J. David Bleich^19^ Rabbi Shlomo Goren^20^
Gestational Development	Maternal status is established through fetal development within the woman’s womb. Rabbi Shaul Yisraeli specifically grounds maternity in the gestational mother because the embryo acquires halachically significant fetal status through development in her womb.	Rabbi Shaul Yisraeli^21^
Birth/Parturition	Parturition establishes a maternal-filial relationship with the woman who gives birth. The possibility that the genetic mother may possess an additional halachic maternal relationship cannot necessarily be excluded.	Rabbi J. David Bleich^19^
Dual/*Safek* Maternity	Because the classical sources do not conclusively establish one exclusive criterion, both the genetic mother and the birth mother should be treated as mothers for relevant halachic purposes (*le-chumra*).	Rabbi Shlomo Zalman Auerbach^23^
IVF-Specific Disruption of Conventional Parentage	Under this distinctive minority approach, fertilization occurring entirely outside the woman’s body may fail to establish ordinary genealogical parenthood. This position should not be classified simply as genetic, gestational, or birth-based maternity.	Rabbi Eliezer Waldenberg^24^

### Fatherhood Status

In contrast to the debate surrounding maternity, halachic authorities generally maintain that paternal identity is determined genetically, assigning fatherhood on the basis of biological paternity (i.e. the sperm donor) (see Table 2). This approach is rooted in the halachic framework in which paternal status determines *kehuna*, *leviyah*, inheritance, and incest laws. Thus, sperm donation creates halachic uncertainty in lineage (*safek yichus*) and incest prohibitions.

**Table 2:** Halachic Approaches to Establishing Paternity.

Approach/Criterion	Main Position	Representative *Poskim*/Sources
Genetic/Sperm Source	The man whose sperm produces the child is generally regarded as the halachic father even when conception occurs without sexual intercourse; assisted reproduction does not by itself eliminate paternal lineage.	Rabbi Moshe Feinstein^25^; Rabbi Shlomo Zalman Auerbach^23^
Non-coital Conception—Question Raised	The absence of intercourse raises a question whether the sperm provider fulfills *pru u’rvu* and whether the resulting child is considered his son “in every respect.” This source raises the doubt rather than establishing an opposing rule of paternity.	Rabbi Moses ben Isaac Judah Lima^26^
IVF-Specific Disruption of Conventional Paternity	Under the minority view that extracorporeal fertilization disrupts ordinary genealogical relationships, the sperm provider may not acquire conventional halachic paternal status through IVF.	Rabbi Eliezer Waldenberg^24^
Social/Raising Relationship—Not Technical *Yichus*	One who raises a child may be regarded as though he fathered the child in an important moral and social sense; this statement does not, by itself, establish technical halachic paternity for lineage, incest, inheritance, or *kehuna*/*leviyah*.	Babylonian Talmud, Sanhedrin 19b^27^

Major *poskim* [rabbinic authorities who issue halachic rulings] such as Rav Moshe Feinstein^25^ and Rav Shlomo Zalman Auerbach^23^ accord substantial halachic significance to biological paternity through the sperm source, although its scope remains debated. The Beis Shmuel^28^ (citing Hagahot Semak as brought by Bach, Yoreh De’ah 195) likewise treats biological contribution outside intercourse as potentially sufficient for parenthood. The Chelkat Mechokek^26^ discusses a woman impregnated by semen in a bathhouse (*nit’abra b’ambati*), asking whether the man fulfills *pru u’rvu* and whether the child is his son in every respect. Chagigah 15a similarly contemplates conception from semen deposited in a bathhouse.^29^ The Alphabet of Ben Sira identifies Jeremiah as Ben Sira’s biological father through such a scenario; however, it does not establish a halachic ruling.^30^

Two biblical verses may offer a conjectural insight into paternal lineage. Genesis 35:24 identifies Joseph and Benjamin as “Rachel’s sons,”^31^ while Genesis 35:26 groups Jacob’s sons as those “born to him in Paddan-aram,” although Benjamin was probably conceived there but born near Bethlehem. While Ibn Ezra on Genesis 35:26 offers a nongenetic explanation of the wording,^32^ we propose that these verses may raise the possibility (not necessarily a proof) that paternal status is linked to conception rather than birth. Megillah 13a states that Esther was parentless because her father died when her mother became pregnant and that her mother died at Esther’s birth.^33^ Whether this aggadic passage bears on technical halachic paternity remains uncertain.

The determination of halachic fatherhood is also relevant to posthumous sperm retrieval and reproduction. A central issue is whether the deceased man is regarded as the halachic father of a child conceived using sperm retrieved after his death. This subject has been discussed extensively in the halachic literature.^34^ For example, Rabbi Shlomo Zalman Auerbach and several other authorities maintain that the biological sperm provider remains the child’s father and that the child therefore retains the deceased’s genealogical lineage.^23^

### Halachic Status of Embryos from More Than Two Parents

In general, halachic parenthood is tied to the contribution of procreative seed, rather than ancillary cellular organelles. The main unprecedented halachic challenge in MRT is that it introduces a third genetic contributor, the woman who donates her mitochondria. Some contemporary halachic authorities argue that mtDNA alone does not establish halachic motherhood,^35^,^36^ because (1) mitochondrial DNA constitutes only a small fraction of the human genome, the vast majority of genetic material being contained within the nuclear chromosomes, and (2) mtDNA does not encode the phenotypic traits typically associated with familial resemblance.

Thus, donation of mtDNA may not confer halachic maternal status on the donor. In contrast, some contemporary halachic authorities raise the possibility that a heritable genetic contribution may have independent halachic significance for familial identity and that the mitochondrial donor cannot simply be dismissed because mitochondrial DNA is critical to life.^12^

Niddah 31a^37^ describes three partners in the creation of every human being: the father, the mother, and the Almighty. The passage continues (author’s translation):

The father “produces seed” contributing the “white” elements of the body, from which the following bodily components are formed: the bones, sinews, nails, the brain, and the white of the eye. The mother contributes the “red” elements, from which are formed the skin, the flesh, the hair, and the black of the eye. And the Almighty endows the person with a spirit, a soul, his facial countenance, power of sight of the eye, power of hearing of the ear, the [capability of] speech of the mouth, the [capability] of walking [with] legs, understanding, and rational faculty.^37^

Discussions of human cloning illustrate the broader interpretive question raised by this Talmud passage. For example, Rabbi Dr Avraham Steinberg argues that a human clone possesses the same halachic status as any other human being and is therefore fully protected by prohibitions such as murder.^38^ Accordingly, it is unclear whether Niddah 31a limits human reproduction to two parental contributors or merely describes the ordinary biological pattern of reproduction. The passage may be read descriptively rather than prescriptively, namely, that a child is ordinarily created through the participation of these three partners, rather than that a child must be created exclusively in that manner. Some traditional sources emphasize a male–female reproductive framework,^39^ while contemporary writers have invoked Niddah 31a in arguing that reproductive arrangements departing from the conventional two-parent model raise distinct halachic concerns.^40^ Experimental primate embryology further complicates assumptions of a one-embryo/oneindividual developmental lineage: viable chimeric rhesus macaques have been generated by combining blastomere aggregates from six four-cell embryos.^41^

Rabbi Moshe Tendler interpreted the Talmudic principle in Niddah 31a through the lens of modern reproductive biology, noting that the father and mother each contribute approximately half of the child’s nuclear genetic material (personal communication, 2014). Rabbi Tendler did not exclude the mitochondrial donor from halachic parenthood; rather, all genetic donors may have some halachic status.^12^ Rabbi J. David Bleich separately discusses Niddah 31a in evaluating modern genetic evidence of parentage.^42^

It is important in this halachic analysis to recognize that, from a medical perspective, mtDNA plays a critical functional role in multiple organ systems. For example, the brain is one of the most energydemanding tissues in the body. Mitochondrial DNA mutations have been linked to several neuropsychiatric disorders, such as schizophrenia, bipolar disorder, and major depressive disorder.^43^,^44^ In addition, skeletal muscle, particularly oxidative type I fibers, is highly dependent on mitochondrial oxidative metabolism.^45^ The high mitochondrial content of type I (“red”) skeletal muscle fibers^45^–^49^ provides an intriguing modern biological parallel, though not a literal equivalence, to the Talmudic attribution of the body’s “red” components to the mother. Consistent with their systemic importance, skeletal-muscle mitochondrial pathways are altered in individuals with neuropathologic Alzheimer’s disease and correlate with disease severity.^50^

There are other halachic concerns about “three-parent reproduction” that are beyond the scope of this paper but need to be fully addressed. As stated above, they include, for example, concerns about the mitzvah of honoring one’s parents,^51^,^52^ the mitzvah of *pru u’rvu*,^53^,^54^ the mitzvah of *yibum*,^55^ *pidyon haben*, 56,57 and the question of *mamzerut*.^58^,^59^

## BIPARENTAL MRT AS A THEORETICAL APPROACH TO SOME HALACHIC ISSUES RELATED TO MRT

Scientists can now reprogram adult somatic cells, such as skin fibroblasts^60^ or peripheral blood cells,^61^ into induced pluripotent stem cells, which can subsequently be directed toward germ cells^62^ and gonadal lineages^63^ through the introduction of defined transcription factors. Most notably, Murakami et al.^64^ demonstrated that induced pluripotent stem cells derived from the tail of an adult male mouse could be converted from an XY to an XX chromosome complement and differentiated into mature, functional oocytes capable of producing offspring after fertilization. Although the generation of fully functional human gametes from somatic cells remains an emerging field, recent developments have extended early proof-of-concept to human cells. In June 2026, the biotechnology company Conception (Berkeley, California, USA) reported generating early human egg cells, described as “primary oocytes” from reprogrammed blood cells.^65^ The company reported that these cells developed within three-dimensional “mini-ovaries” containing germ cells and ovarian-supporting cells, including structures resembling human primordial follicles. However, these cells are not yet mature, fertilization-competent oocytes. All these advances represent important developments in human cell-derived gametogenesis, but they have not yet established a clinically functional human gamete-generation system. Further progress requires the coordinated recreation of germ-cell specification, gonadal development, epigenetic remodeling, sexual differentiation, and meiosis progression.^66^

Although stem cell-derived gametogenesis has not matured into an established human reproductive technology, we propose a theoretical application to MRT. Specifically, induced pluripotent stem cells derived from the husband’s somatic cells (containing healthy mtDNA) would need to be generated and differentiated into an oocyte-like cytoplasmic host cell capable of supplying mitochondria and the cellular environment required for subsequent nuclear transfer and fertilization. We hypothesize that, if such a male-derived host cell could be generated with appropriate cytoplasmic architecture, mitochondrial content, epigenetic state, and developmental competence, it could serve as the basis of the proposed biparental mitochondrial replacement therapy (Bi-MRT) strategy (Figure 1). The proposed protocol would also require confirmation that the husband-derived mitochondrial population lacks clinically significant pathogenic variants and that reprogramming and differentiation preserve mitochondrial function. This proposal therefore depends on capabilities that have not yet been demonstrated in a clinically usable human system.

**Figure 1:**
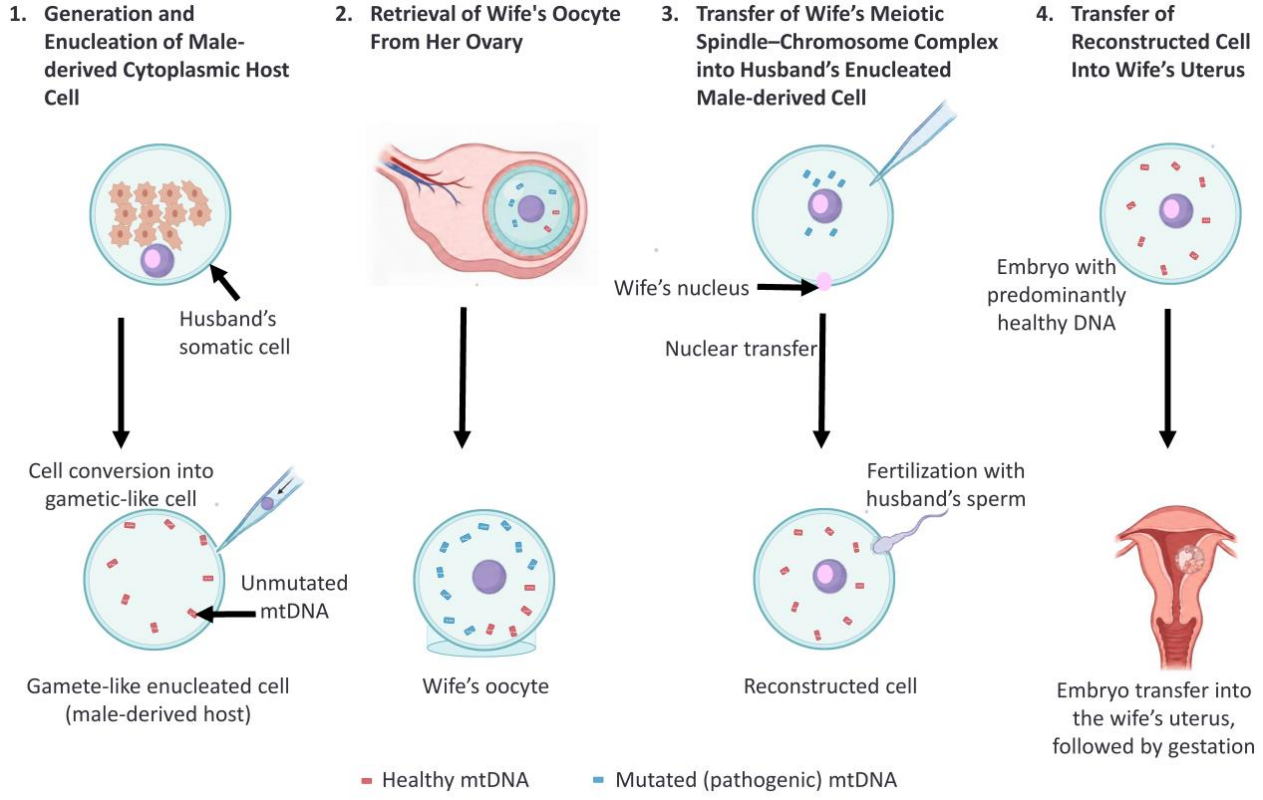
Biparental MRT—A Theoretical Protocol to Reduce Transmission of Pathogenic mtDNA Variants. Gemini AI was used solely to assist in generating the illustrative artwork.

It is critical to note that sourcing the cytoplasmic host from the husband specifically is a targeted halachic and psychosocial choice designed to restore the traditional two-parent order; it is not a technical limitation of the reprogramming technology itself. Technically, one could use the father’s gamete-like cell without enucleation and then fertilize it with the father’s sperm, creating a virtual human clone of the father. Similarly, the father’s gamete could be fertilized with another man’s sperm. From a halachic perspective, these reproductive configurations might conflict with an interpretation of the Talmud’s principle^37^ that children are created from a man and a woman. Rabbi Chaim Jachter reports an argument that human cloning is in tension with the Biblical-Talmudic model of reproduction described in Niddah 31a, which identifies God, father, and mother as the three partners in human creation.^67^

It is important to emphasize that if mtDNA is halachically insignificant, then the rationale for using the husband’s cytoplasm may require further clarification. Nonetheless, sourcing cytoplasm from the husband is not a strict legal requirement but serves as an additional psychosocial and halachic safeguard. Utilizing paternal cytoplasm can eliminate third-party donor involvement, preserve an exclusively bilateral marital framework, and perhaps circumvent lingering halachic doubt (*safek yichus*).

We therefore propose that the biological, functional, and heritable characteristics of mtDNA provide a basis for considering mtDNA donation as having independent halachic significance in determining parenthood. Under the principal geneticpaternity approach discussed above, paternal status is anchored by the husband’s sperm contribution. 22,24 Meanwhile, Bi-MRT unites the principal proposed criteria for maternity, i.e. the wife’s nuclear genetic contribution, gestation, and parturition, within a single individual. Assigning functional or halachic significance to paternally derived mtDNA consolidates all genetic contributions within the married couple, thereby transforming a potential “three-parent” genetic arrangement into the more straightforward halachic framework of two-parent reproduction without affecting maternal identity, while preserving a bilateral genetic relationship between the husband and wife.

While significant questions regarding chromatin stability and epigenetic resetting remain unresolved, this protocol may introduce a paradigm shift from a halachic perspective. Biparental MRT could theoretically transform the operational framework of MRT from a three-parent genetic matrix to a traditional, bilateral two-parent model. While this type of procedure is not yet ready for human clinical trials, we suggest that rabbis consider the halachic parameters of Bi-MRT as a framework that may help address some of the halachic challenges associated with conventional MRT.

One important issue requiring clarification concerns the child’s halachic status when the husband is not halachically Jewish in our Bi-MRT protocol. Here, the woman contributes only her nuclear DNA and not mtDNA, whereas the husband is contributing his nuclear DNA and the cytoplasmic and mitochondrial components of the gamete. If a non-Jewish husband is involved in Bi-MRT with his Jewish wife, will the child be Jewish? Rabbinic authorities must therefore determine whether matrilineal descent is established by the woman’s nuclear genetic contribution, or whether halachic maternity also depends upon the origin of the oocyte cytoplasm and mtDNA.

Rabbi Asher Weiss regards the determination of halachic maternity in cases of egg donation and surrogacy as unresolved by the classical sources. Although his inclination is that the woman who provides the oocyte is the halachic mother, he does not consider this conclusion sufficiently certain to exclude the gestational mother from halachic consideration; accordingly, he treats the child as Jewish for other halachic purposes while nevertheless recommending conversion *le-chumra* [as a precaution] because of the possibility that the gestational surrogate has maternal status.^68^

In the distinct context of mitochondrial replacement therapy, however, Rabbi Weiss places decisive weight on the woman who contributes the oocyte nucleus and the overwhelming majority of the child’s genetic material. Accordingly, when that woman is Jewish and the mitochondrial donor is non-Jewish, he concludes that the child is Jewish for all halachic purposes.^36^ His analysis is therefore particularly relevant to Bi-MRT, in which nuclear, cytoplasmic, and mitochondrial contributions are allocated between the spouses. A related question concerns paternal status. Rabbi Moshe Feinstein maintained, in the context of artificial insemination, that the genetic contribution of the husband may establish halachic paternity despite the absence of sexual intercourse.^25^ Other authorities have taken more restrictive approaches to the establishment of halachic parenthood through assisted reproductive technologies. Rabbi Eliezer Waldenberg, for example, expressed substantial reservations regarding the attribution of conventional halachic parenthood in certain forms of extracorporeal conception,^24^ and other *poskim* have likewise debated whether genetic contribution alone, in the absence of sexual intercourse, is sufficient to establish parental status.^69^ These divergent rabbinic approaches underscore the need to determine separately how nuclear genetic contribution, cytoplasmic contribution, gestation, and birth should be weighted in defining halachic parenthood in Bi-MRT.

Another concern relates to the rapid development of CRISPR (clustered regularly interspaced short palindromic repeats) technology and its potential application to MRT. If therapeutic genes from a third party were introduced into an embryo through CRISPR-mediated genetic sequences to correct pathogenic mutations, such as those associated with Tay–Sachs disease, Canavan disease, or hereditary breast cancer, we suggest that the donor of such a limited therapeutic sequence would not thereby acquire halachic parental status. This analogy suggests that the mere contribution of limited genetic material is insufficient to establish halachic parenthood. Halachic parenthood does not require that every molecule of DNA or genetic sequence incorporated into a developing individual confer parental status. If it did, many forms of gene therapy that introduce exogenous genetic material would generate additional claims of parenthood, a conclusion for which there is no evident halachic basis. Biparental MRT may be conceptually distinct because it introduces an entire population of mitochondria carrying a complete and potentially heritable mitochondrial genome, rather than a limited therapeutic DNA sequence. Nevertheless, whether this difference has halachic significance remains open to debate. If mtDNA is accorded independent halachic relevance in establishing parenthood, then a third-party mitochondrial donor could raise a genuine question of maternal status. Conversely, if mtDNA is viewed as biologically important but halachically ancillary, the mitochondrial donor would be more appropriately understood as a biological facilitator of therapy rather than a parent. Biparental MRT would eliminate the specific question of a third-party mitochondrial donor by sourcing the mitochondria from the husband. Because the husband also contributes the sperm, all paternal genetic contributions would originate from a single individual, providing the principal basis for halachic paternal status.

Crucially, although Bi-MRT is intentionally designed to preserve the conventional two-parent reproductive framework, the underlying cellular reprogramming technologies could, at least conceptually, enable non-traditional reproductive configurations, including uniparental androgenesis and male-derived mitochondrial donation. From a halachic perspective, the permissibility of initiating such novel reproductive arrangements must be evaluated independently of questions concerning the resulting child’s legal status, lineage, and parental identity. Moreover, recognizing a functional role for male-derived mtDNA in Bi-MRT does not alter the principal proposed determinants of halachic maternity. The strongest conventional maternal claims remain grounded in the wife’s nuclear genetic contribution, gestation, and parturition, although the independent halachic significance of the cytoplasmic and mitochondrial source remains unresolved. Consequently, the husband’s mitochondrial contribution serves as a therapeutic biological component rather than a basis for maternal attribution.

## PSYCHOSOCIAL/HALACHIC ADVANTAGES OF BIPARENTAL MRT

Aside from its proposed halachic rationale, Bi-MRT may also have psychosocial implications. Couples may prefer to reproduce using their own gametes to preserve a shared genetic connection with the child and avoid complexities associated with a third-party genetic contributor.^70^ Research on donor conception indicates that genetic relatedness and parent–child resemblance can have psychological and relational significance, particularly for the non-genetic parent.^71^ Although donor-conceived children generally demonstrate normal psychological adjustment, the absence of a genetic link may raise questions concerning resemblance and identity.^72^ Disclosure practices can also influence family dynamics and the experiences of donor-conceived individuals.^73^,^74^ These findings derive primarily from studies of conventional donor conception and therefore should not be assumed to apply directly to mitochondrial donation. Nevertheless, emerging research has begun to examine psychosocial attitudes toward MRT. For example, Noohi et al.^75^ reported that seven of eight participants affected by mitochondrial disease expressed a preference for having children genetically related to both intended parents. The investigators also found that participants generally did not regard mitochondrial donors as third parents, distinguishing genetic contribution from social parenthood. These findings suggest that genetic relatedness and perceptions of parental identity may influence reproductive decision-making in the context of MRT. Although it remains uncertain whether Bi-MRT would confer measurable psychological benefits, eliminating third-party mitochondrial donation could potentially reduce some donor-related identity and relational concerns. This potential advantage, however, remains speculative and requires further empirical investigation.

## CONCLUSION

This paper explores the complex intersection of cutting-edge genomic bioengineering and the rigorous framework of halacha. Although conventional mitochondrial replacement therapy (MRT) may represent a major clinical advance for women carrying pathogenic mtDNA mutations, its “three-parent” structure introduces a third genetic contributor. This feature challenges traditional conceptions of parenthood and may create both psychological concerns and a halachic uncertainty regarding lineage (*safek yichus*) for religious families. We therefore propose biparental mitochondrial replacement therapy (Bi-MRT) as a theoretical scientific approach that may address many of the halachic concerns associated with conventional MRT. By preserving a bilateral genetic connection to the married couple, this “two-parent” MRT model may provide a framework for addressing central questions of motherhood, parenthood, and lineage, while reducing donor-related identity concerns. In particular, Bi-MRT may lessen uncertainty regarding halachic maternity by eliminating the need for a third-party female mitochondrial donor and uniting the wife’s nuclear genetic contribution, gestation, and parturition within a single individual. As genomic technologies continue to advance, Bi-MRT offers a potential conceptual model for pursuing therapeutic innovation while preserving the coherence of traditional halachic notions of family and lineage.

## Abbreviations

DNA: deoxyribonucleic acid
IVF: *in vitro* fertilization
MRT: mitochondrial replacement therapy
mtDNA: mitochondrial DNA
